# Clicked H-Shaped Arylopeptoids

**DOI:** 10.3390/molecules30030724

**Published:** 2025-02-05

**Authors:** Zein El Abidine Chamas, Ayman Akhdar, Florence Charnay-Pouget, Sophie Faure, Arnaud Gautier

**Affiliations:** Université Clermont Auvergne, Clermont Auvergne INP, CNRS, ICCF, F-63000 Clermont-Ferrand, France; zein.chamas@uca.fr (Z.E.A.C.); ayman.akhdar@hotmail.com (A.A.); florence.charnay_pouget@uca.fr (F.C.-P.); sophie.faure@uca.fr (S.F.)

**Keywords:** *N*-alkylated aromatic oligoamides, arylopeptoids, CuAAC, Copper(I) *N*-heterocyclic carbene, solid-phase synthesis, cross-ligation

## Abstract

This study presents a tentative synthesis of supported H-shaped and ladder-type compounds. If the ladders were not accessible, probably due to distance misfits between the reactive centers, a facile method for the synthesis of H-shaped *N*-alkylated aminomethyl oligobenzamides, i.e., arylopeptoids by on-resin homodimerization via the Copper(I)-Catalyzed-Alkyne-Azide-Cycloaddition (CuAAC) reaction is reported. While successful, a synthetic bottleneck was identified for further oligomer elongation due to congestion when the ligation occurs on solid support. However, this issue was effectively addressed using an elongated oligomer to conduct inter-strand cross-linking. Further CuAAC functionalization could be performed after elongation with additional alkyne groups to enhance diversity.

## 1. Introduction

Access to aromatic oligoamides with specific shapes has become increasingly valuable for creating well-defined architectures with novel properties [1,2,3,4,5]. The decoration of such edifices is also of particular interest for conceiving multivalent ligands or inhibitors [6]. It is therefore important to develop facile and straightforward methodologies to access polyfunctionalized architectures of defined sequences. Bioconjugation and ligation techniques are particularly appropriate for solid-phase synthesis, often using automated protocols to modify synthetic oligonucleotides and peptides [7,8,9]. Among these, Copper(I)-Catalyzed Alkyne-Azide Cycloaddition (CuAAC) is especially popular due to its modularity, regiospecificity, high yield, straightforward reaction conditions, and maximum atom economy [10,11,12,13]. However, its experimental conditions require special attention to the solid support. Selecting solvents that facilitate both the reaction and adequate swelling of the matrix is crucial. Additionally, the reaction temperature (achieved by heating with a jacketed reactor or using microwave irradiation [14,15]) is typically higher than that used for reactions conducted in solution. Various copper(I) sources have been reported to catalyze supported reactions, including copper(I) iodide (CuI), copper(I) bromide (CuBr), copper(II) sulfate (CuSO_4_), and metallic copper (Cu(0)).

The most common method, reported by Meldal, utilizes CuI and *N,N*-diisopropylethylamine (DIPEA) in *N,N*-dimethylformamide (DMF) as the solvent at 25 °C [11]. Additionally, the use of a reducing agent (such as sodium ascorbate) to convert copper(II) sources into copper(I) in situ was reported by Kirshenbaum for supported peptoid synthesis [16]. It is important to note that, in many cases, copper catalysts are used in at least equimolar amounts, often with significantly large quantities of catalyst; for instance, 7 equivalents of ascorbic acid, 13 equivalents of CuI, and 17 equivalents of DIPEA. The application of CuAAC is thus challenging due to the high amounts of copper and reducing agents required. In addition, reports have indicated that active copper species can form radicals that partially degrade functionalized groups during CuAAC reactions [17]. Finally, for in vitro systems, residual copper may also interfere with cellular metabolism [18,19]. Therefore, to address these challenges, many systems have been proposed [20,21,22,23]. In this context, we introduced *N*-heterocyclic copper(I) carbenes as catalysts, which can be used in lower catalytic amounts (5 to 10 mol%) [24,25].

**Scheme 1:**
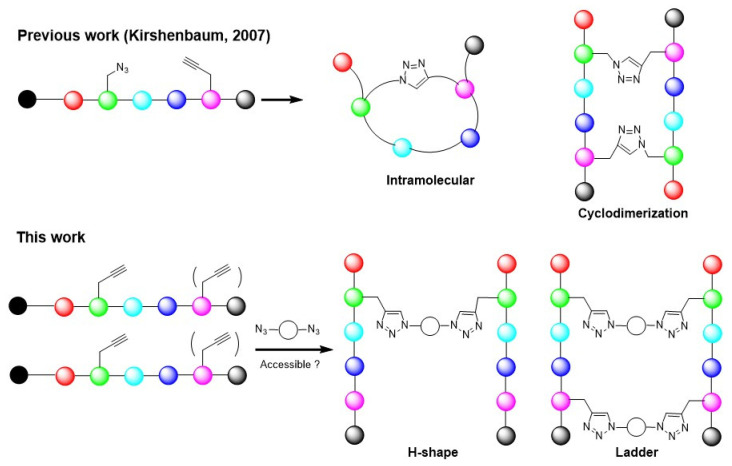
Intra- and inter-strand CuAAC ligation (the black circle represents the solid support, and the white circle represents the central moiety of the diazide) [29].

In a previous project on the design of polyfunctionalized *N*-alkylated aromatic oligoamides [26], our group reported that supported *N*-substituted aminomethyl benzamide oligomers named arylopeptoids containing two or more alkyne side chains, could serve as excellent substrates for on-resin click reactions using air-stable copper(I) *N*-heterocyclic carbene catalysts (Cu(I)-NHC) to access functionalized aromatic oligoamides [27]. In this context of macrocyclic oligoamide synthesis on support, Finn and Kirshenbaum’s groups demonstrated that supported peptide or peptoid oligomers containing a single pair of alkyne and azide groups undergo intramolecular cyclization or cyclodimerization using copper iodide and sodium ascorbate as catalysts, to form macrocycles with the ratio depending on the preorganization of reactive groups along the oligomers adopting helix or β-sheet secondary structure (Scheme 1) [28,29].

These findings suggest that inter-strand linkage can occur on the solid support, indicating that larger structures of defined sequence could be formed through a minimum of synthesis steps and a simple ’click-dimerization’ process. Therefore, we hypothesized that supported arylopeptoid oligomers with a single alkyne would react with a bis-azide in solution to produce a symmetric H-shaped structure, as shown in Scheme 1. Additionally, a supported substrate containing multiple alkynes would yield ladder-like structures.

## 2. Results and Discussion

**Scheme 2:**
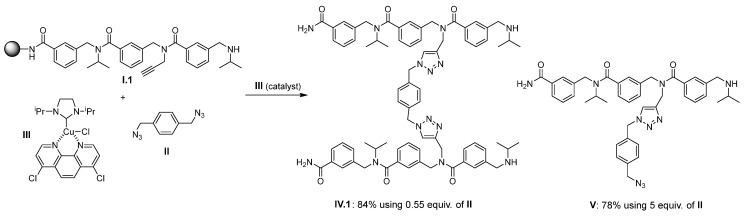
CuAAC catalyzed inter-strand ligation (the black circle represents the Rink Amide resin).

**Figure 1:**
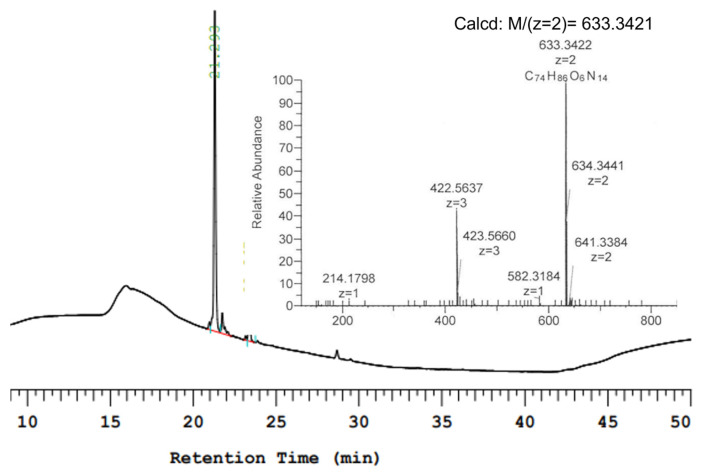
HPLC trace and mass spectrum of compound **IV.1**.

To explore the occurrence and efficiency of the on-resin homodimerization of arylopeptoid oligomers by a double click process, we began the experiment by testing the reaction of the *meta*-arylopeptoid trimer **I.1** supported on Rink Amide resin. This compound contains one alkyne side chain on the second residue. It is important to note that the resin used in the present study is a Rink amide resin (100–200 mesh) with a loading capacity of 0.62 mmol/g. This was chosen to meet the optimal conditions for obtaining triazole-containing arylopeptoids previously reported [27] and to ensure good yields of the supported starting material bearing alkyne(s). The supported *meta*-arylopeptoid trimer **I.1** was first synthesized using the well-established submonomer protocol involving an acylation step using 3-chloromethylbenzoylchloride followed by a substitution step with isopropyl or propargyl amine (see experimental section for more details). It was then placed in the presence of 0.55 equivalents of 1,4-bis(azidomethyl)benzene (**II**), and the reaction was catalyzed by the copper(I)-*N*-heterocyclic carbene complex **III** (Scheme 2). We chose the diazide **II** due to its relatively low rotational freedom compared to alkyl analogs. Concerning the copper (I) catalyst, it was previously demonstrated that NHCs-Cu(I), especially the complex [(SIMes)(4,7-dichloro-1,10-phenanthroline) CuCl], outperform classical systems commonly used in peptide modification [30,31] and arylopeptoid modification [32]. This system is fully soluble in many organic solvents, insensitive to air oxidation, presents thermal stability, and provides higher turn-over, allowing its use in catalytic amounts [25]. The catalyst has been characterized as a tetrahedral copper complex in the solid state. In solution, the phenanthroline moiety facilitates the dissociation of the chloride ion, forming a cationic species with enhanced reactivity. While the catalyst performs well at room temperature for reactions conducted in solution, we have reported that solid-phase synthesis requires higher temperatures. The best solvent identified so far is a mixture of methanol and dichloromethane (DCM). Methanol is necessary to ensure proto-decupration at the final stage of the catalytic cycle, while DCM is essential to achieve adequate resin swelling. Therefore, the catalyst was employed at 10 mol% at 50 °C in a MeOH/CH_2_Cl_2_ mixture (*v*/*v* 2:8), providing a full conversion of the starting material **I.1** that was observed after 4 h. Upon cleavage with TFA/H_2_O/TIS (95: 2.2: 2.5), we were pleased to isolate the anticipated H-shaped structure **IV.1** as the major product with an 84% isolated yield and 90% UV purity using HPLC (Figure 1). Notably, this isolated yield is consistent with the reported yields for synthesizing the linear clicked arylopeptoid (70–90%) [27]. The ^1^H NMR spectrum of IV.1 (see Supplementary Materials) displays a series of broad signals due to the presence of multiple *N*,*N*-substituted amide rotamers, which interconvert at a rate comparable to the NMR timescale. Conversely, when a large amount of diazide **II** (5 equivalents, 10 times more than for the previous experiment) was used, the homodimerization process was not efficient, and a mixture of the mono-clicked compound **V** and the dimeric compound **IV.1** in an 8:1 ratio (HPLC) was obtained. The mono-clicked compound **V** was isolated with 78% yield and 97% UV purity (HPLC). This observation highlights that the cross-linking process is not very rapid and that the reaction outcomes can be easily tuned. Although it falls outside the scope of this report, the successful synthesis of mono-clicked oligomer V in an acceptable yield suggests that this compound could serve as a starting material for preparing unsymmetrical H-shaped compounds.

**Scheme 3:**
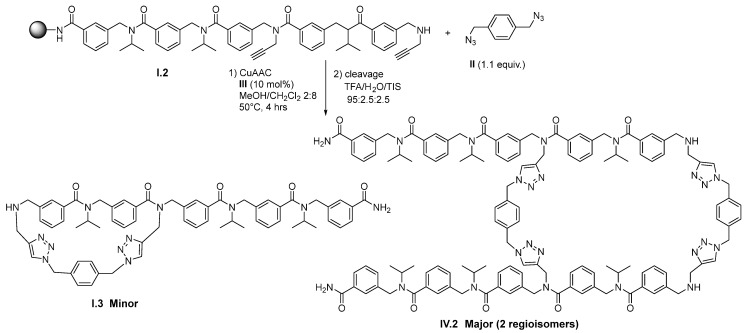
Attempt of formation of ladder-type oligomer (the black circle represents the Rink Amide resin).

To explore the possible formation of a ladder-type structure, we examined the behavior of the supported arylopeptoids containing two alkynes, as depicted for the pentamer **I.2** (Scheme 3). It is important to note that using this supported compound, the CuAAC reaction can occur randomly at the two positions. Unfortunately, the reaction conditions (0.55 equivalents of 1,4-bis (azidomethyl) benzene **II** per alkyne and 10 mol% of Cu(I)-NHC **III**) failed to produce a single product but instead resulted in the inter-stand cross-linked oligomer **IV.2** as the major products. This compound was accompanied by the formation of cycloarylopeptoid **I.3** from an intra-strand ligation as a minor product (identified by HR-MS). The presence of numerous polymeric compounds as minor components complicates the purification process and the presence of the two regioisomers of the cross-linked compound **IV.2**, prompting us to set aside this method.

**Scheme 4:**
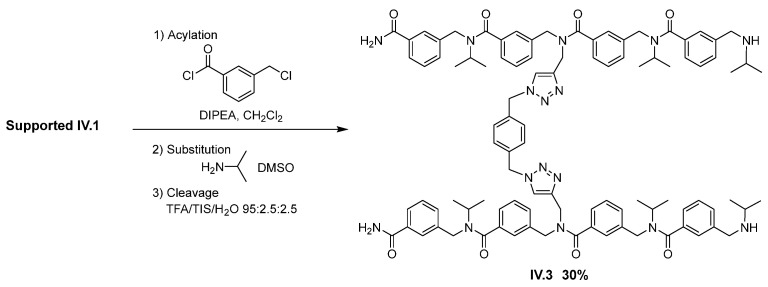
Attempt of H-shaped arylopeptoid elongation.

According to our first experiment, the homodimerization process appears very efficient when using 0.55 equivalents of bis-azide. However, in the case of multiple cross ligations to form a ladder, regioisomers formation can occur. Indeed, the inter-strand double-click reaction can proceed between alkynes on the same residue or on different residues. It would therefore be more suitable to build each rung of the ladder sequentially (vide infra). We thus tested the possible elongation of the H-shaped compound on solid support. Notably, this strategy could allow the introduction of additional alkynes for further click functionalization(s). However, the reaction of supported arylopeptoid **IV.1** with 3-chloromethyl benzoyl chloride, followed by substitution with isopropyl amine using a well-established protocol [27,33], encountered difficulties, yielding only trace amounts of the desired compound **IV.3** (Scheme 4). A modest improvement was achieved by employing harsher conditions (6 equivalents of 3-chloromethyl benzoyl chloride and extending the reaction time to one hour, repeated twice), leading to a 30% isolated yield of the targeted compound **IV.3** (94% UV purity). Interestingly, the only other isolated compound (50–60%) was the unreacted starting material, indicating that the acylation step has become a synthetic bottleneck.

**Scheme 5:**
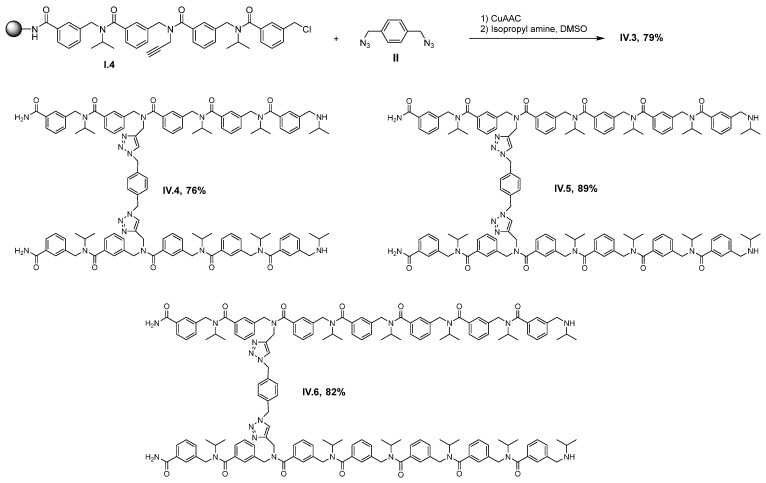
Improved strategy for H-shaped arylopeptoid elongation (the black circle represents the Rink Amide resin).

We hypothesized that the problematic post-linkage acylation is due to steric congestion between the two strands after the CuAAC reaction. To address this issue, we tested the homodimerization on the longer-supported tetramer **I.4** containing a chlorobenzyl group at the terminal position. This was followed by a reaction with isopropyl amine on the linked compound. We were delighted to obtain the desired compound **IV.3** in a 79% isolated yield and 94% HPLC purity. The success of this ’click-then-elongate’ strategy confirms that steric hindrance is the primary factor to consider. Interestingly, this improvement, which rapidly generates a structure containing eight residues, confirms that the click reaction is unaffected by the presence of the reactive benzyl chloride functional group. Therefore, the method of ‘click then elongate’ was successfully extended to synthesize symmetrical dimeric oligomers **IV.4**–**IV.6** with up to 14 residues, yielding compounds with up to 89% isolated yield and excellent purities ranging between 91 and 99% (Scheme 5).

**Scheme 6:**
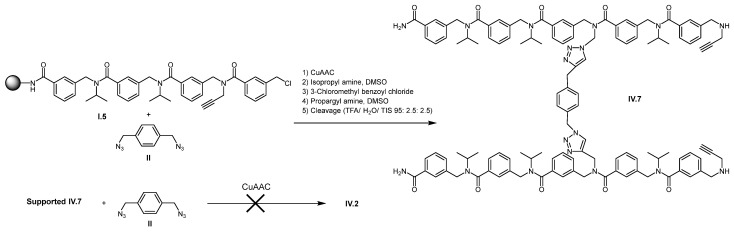
Alkyne side chain installation on the cross-linked oligomer (the black circle represents the Rink Amide resin).

The successful result prompted us to think that elongation could also introduce an additional alkyne. Therefore, we hypothesized that this strategy of ‘click then elongate’ could increase the diversity after additional CuAAC reactions or afford a ladder-type compound in a sequential click sequence, as mentioned before. As depicted in Scheme 6, we try to synthesize **IV.2**. However, the reaction of supported **IV.7** with 0.55 equivalents of bis-azide **II** per alkyne, in the same conditions as previously described, did not afford ladder-type compound **IV.2**, but instead yielded unidentified polymeric compounds, ruling out this route for forming ladder-type structures. This suggests that the distance between the two alkynes has increased, preventing them from accommodating the reaction with bis-azide **II**.

**Scheme 7:**
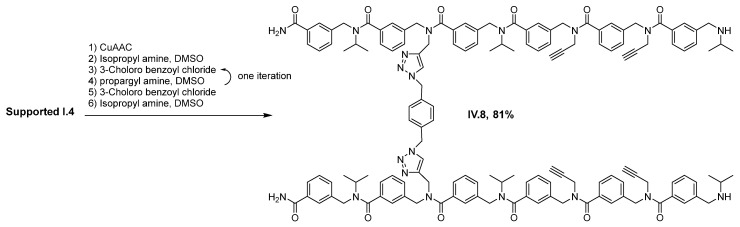
Synthesis of a H-shaped polyalkynated substrate.

However, our ‘click then elongate’ technique can be applied to introduce several propargyl amine side chains at different positions, as depicted in Scheme 7. Using this approach, we successfully extended supported **I.4**, yielding the symmetrical compound **IV.8** with an excellent isolated yield of 81%.

**Scheme 8:**
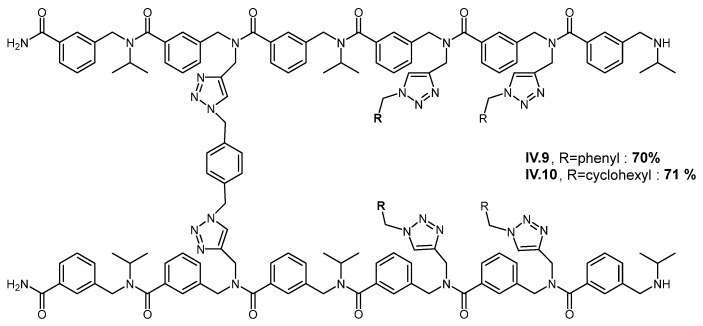
Diversely substituted H-shaped oligomers.

Supported **IV.8** can be used as a starting material to expand the scope for synthesizing diverse substituents via the CuAAC reaction, this time using a mono-azide reagent. Following the execution of a second on-resin CuAAC reaction with benzyl or methyl cyclohexyl azide on **IV.8**, we successfully isolated the anticipated triazoles **IV.9** and **IV.10** with good isolated yields (70 and 71%) and purities (Scheme 8). The successful reaction of mono-azides with bis-azide **II**, leading to the formation of a polymeric compound, demonstrates that CuAAC is feasible. However, the distance between the two reactive centers in the preformed supported H-shaped compound must be considered when forming a ladder-type compound.

In conclusion, we reported the facile synthesis of H-shaped arylopeptoids through a double CuAAC reaction catalyzed by a Cu(I)-NHC with a bis-azide, utilizing a supported substrate containing an alkyne side chain. Our study identified a synthetic bottleneck caused by congestion when the cross-linking bis-triazole is positioned near the reactive center, particularly evident on the solid support. This limitation was effectively overcome by employing one step further elongated oligomer for the inter-strand ligation by CuAAC reaction, enabling straightforward access to larger structures. This methodology paves the way for the creation of diverse molecular architectures with tailored functionalities, for example, the generation of molecular platforms decorated with several copies of the same ligand or various ligands or the design of complex structures with amphiphilic properties, which could be further explored for their associative and/or antibacterial properties. The potential of these polytriazole aromatic oligoamides is currently under investigation in our laboratory, and results will be reported in due course.

## 3. Materials and Methods

### 3.1. General Information

Chemicals: Rink amine resin (0.62 mmol/g loading, 100–200 mesh) was obtained from Novabiochem (Les Ulis Courtaboeuf, France); piperidine and dichloromethane from Carlo Erba (Les Ulis Courtaboeuf, France); 3-chloromethylbenzoylchloride, TFA, DIPEA, isopropyl, and propargyl amines from TCI; *N*-methylpyrrolidone from Alfa Aesar (Les Ulis Courtaboeuf, France) and DMSO from Acros (Les Ulis Courtaboeuf, France). 1,4-bis (azidomethyl), benzene **II** (azidomethyl), cyclohexane, and benzyl azide were synthesized according to literature procedures [33,34]. For solid-phase synthesis, 10 mL jacketed reactors were purchased from Kamush (Szczecin, Poland) and thermo-regulated using a Lauda thermostat (Lauda, Lauda-Königshofen, Germany). NMR spectra in CDCl_3_ were recorded on a Bruker advance 400 spectrometer (Bruker, Billerica, MA, USA). Purification was performed on a Buchi Pure Chromatography system. High-resolution mass spectra (HRMS) were recorded using electrospray ionization in positive mode (ESI^+^) on a Q Exactive Quadrupole-Orbitrap Mass Spectrometer (Thermo Scientific, Waltham, MA, USA). Liquid chromatography–mass spectroscopy (LC-MS) was recorded on a Q Exactive Quadrupole-Orbitrap Mass Spectrometer coupled to a UPLC Ultimate 3000 (Thermo Scientific, Waltham, MA, USA) equipped with a DAD UV/VIS 3000 RS detector (Thermo Scientific, Waltham, MA, USA)) using a Kinetex EVO C18 (Phenomenex, Torrance, CA, USA); 1.7 µm; 100 mm × 2.1 mm column with a flow rate of 0.45 mL.min^−1^ and the following gradient: a linear gradient of solvent B from 5% to 95% over 7.5 min (solvent A = H_2_O + 0.1% formic acid, solvent B = acetonitrile + 0.1% formic acid). Analytical HPLC was recorded on a Hitachi liquid chromatograph (Oven 5310, 30 °C; Pump 5160; DAD detector 5430, (Tokyo, Japan)) equipped with a C18 Acclaim column (4.6 mm × 250 mm, 5 μm, 120 Å). The detection wavelength was 240 nm or 280 nm, and the flow rate was 0.5 mL/min. Gradient elution used (A) water/0.1% TFA; (B) methanol according to Method A: (Solvents A/B: 95:5 (0–5 min); gradient 95:5 to 5:95 (5–25 min); 5:95 (25–35 min); or Method B (Solvents A/B: 95:5 (0–5 min); gradient 95:5 to 75:25 (5–10 min); gradient 75:25 to 40:60 (10–50 min); gradient 40:60 to 5:95 (50–65 min); 5:95 (65–70 min).

### 3.2. Protocols for On-Resin Arylopeptoid Oligomers Synthesis

Synthesis of meta-arylopeptoid oligomers by submonomer method: All syntheses were performed on Rink amide resin (100 mg, loading 0.62 mmol/g). After swelling in CH_2_Cl_2_ (2 mL) at room temperature for 10 min, Fmoc deprotection was performed by first washing the resin with NMP (*N*-methyl-2-pyrrolidone) (5 × 2 mL) and then the addition of a mixture of piperidine/NMP 1:4 (1 mL). After 2 min of agitation, the resin was drained. Further, a piperidine/NMP 1:4 mixture (1 mL) was added, and the resin was agitated for 15 min, drained, and washed with NMP (5 × 2 mL) and then CH_2_Cl_2_ (5 × 2 mL). Acylation was performed using 3-chloromethylbenzoylchloride (3 equiv. per mmol loading) and DIPEA (6 equiv. per mmol loading) dissolved in CH_2_Cl_2_ (1 mL). After shaking at room temperature for 10 min, the resin was washed with CH_2_Cl_2_ (5 × 2 mL) and DMSO (5 × 2 mL). The substitution was performed using isopropyl or propargyl amine (20 equiv. per mmol loading) dissolved in 0.5 mL of DMSO. The temperature was raised to 50 °C for 1 h; then, the resin was washed with DMSO (5 × 2 mL) and CH_2_Cl_2_ (5 × 2 mL). Acylation and substitution were repeated to grow the targeted arylopeptoid oligomer until the expected sequence length.

On-resin double-click reaction protocol: Resin-bound arylopeptoid trimers obtained from 100 mg of resin were introduced in a reactor containing 1 mL of CH_2_Cl_2_/MeOH mixture (*v*/*v* 8:2). 1,4-bis(azidomethyl)benzene **II** (0.55 equiv. per alkyne or 5 equiv. per alkyne), and 5 or 10 mol% of catalyst [(SIMes)(4,7-dichloro-1,10-phenanthroline) CuCl] **III** per alkyne were added. The reactor was gently shaken for 4 h at 50 °C. The resin was washed with MeOH (5 × 2 mL) at 50 °C and then with CH_2_Cl_2_ (5 × 2 mL) at room temperature.

Cleavage from the resin protocol: The resin was gently shaken in a 1 mL TFA/TIS/H_2_O (95:2.5:2.5) solution for 10 min at room temperature. The solution was drained out and evaporated to dryness under reduced pressure. The product was purified using Buchi LC automatic C18 column using water + 0.1% TFA and MeOH as solvents.

## Data Availability

The authors declare that all data supporting the findings of this study are available within the article or the Supplementary Materials File.

## Supplementary Materials

The following supporting information can be downloaded at https://www.mdpi.com/article/10.3390/molecules30030724/s1, Characterization data (LCMS, HRMS and NMR spectrum) for all synthesized compounds.
