# Supplementary material for: Clicked H-Shaped Arylopeptoids
Source: Molecules. 2025 Feb 5;30(3):724. doi: 10.3390/molecules30030724 (PMC11821099; doi:10.3390/molecules30030724)

Supporting Information

Clicked H-Shaped Arylopeptoids.

Zein El Abidine Chamas, Ayman Akhdar, Florence Charnay-Pouget, Sophie Faure, Arnaud Gautier

Content

Compounds characterization: 2

Compound **II**: 2

Compound **IV.1**: 3

Compound **V**: 4

Compound **IV.3**: 5

Compound **IV.4**: 7

Compound **IV.5**: 8

Compound **IV.6**: 9

Compound **IV.8**: 11

Compound **IV.9**: 12

Compound **IV.10**: 13

Protocols and Compounds Characterization:

1,4-bis(azidomethyl)benzene II:

10 g of 1,4-bis(chloromethyl)benzene (57 mmol, 1.0 equiv.) are dissolved in 50mL of DMSO. 12 g (185 mmol, 3.2 equiv.) of NaN_3 are added under stirring and the solution is heated at 45°C for 72h. After cooling to room temperature, the solution was poured into 200 mL of water and extracted with 200 mL of diisopropyl ether. The organic layer was washed with water (2 X 150 mL), brine (15 mL) and dried over $MgSO_4$. Evaporation affords 10.5 g (98%) of compound **II** as a colourless oil which turn solid on standing.

^{1}H NMR (400 MHz, $CDCl_3$): δ (ppm) = 7.31 (s, 4H), 4.40 (s, 4H).

^{13C} NMR (100 MHz, $CDCl_3$): δ(ppm) = 135.5, 128.5, 54.3.

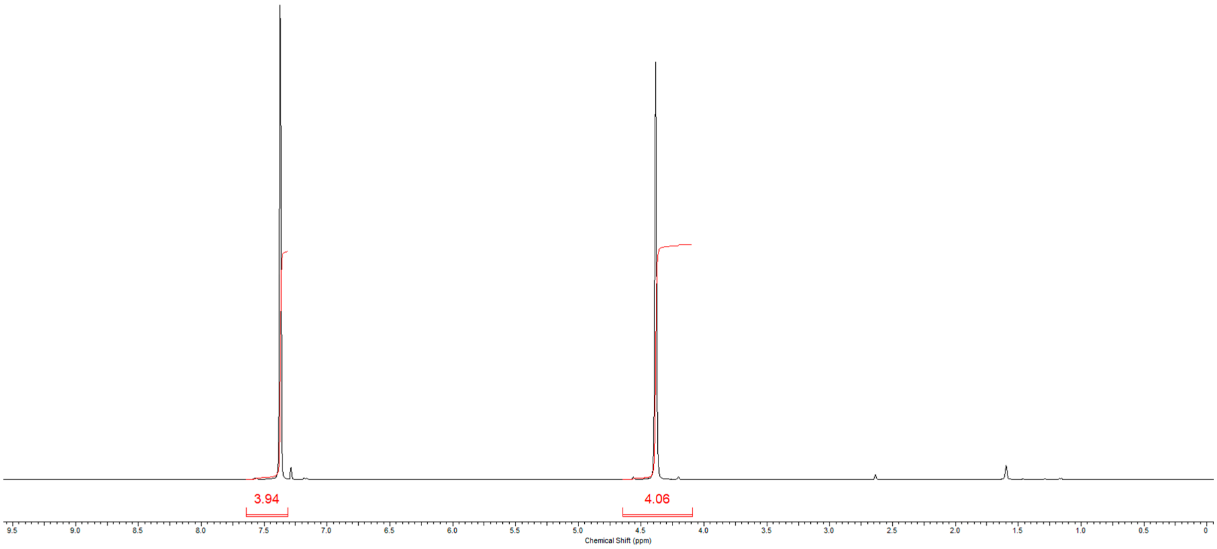

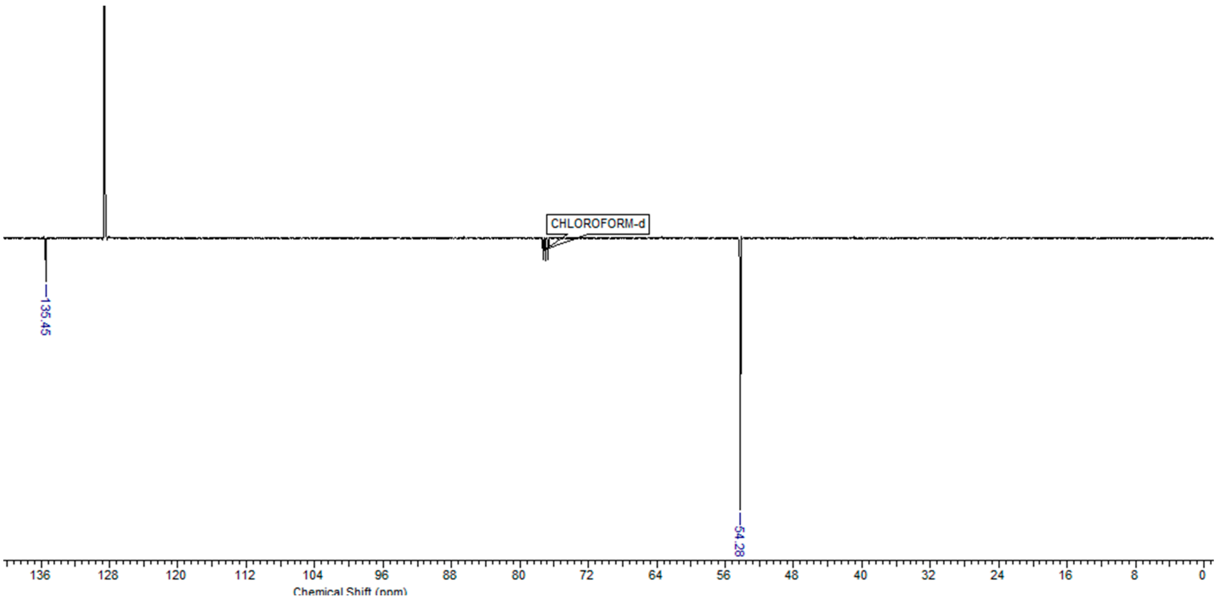

Compound IV.1:

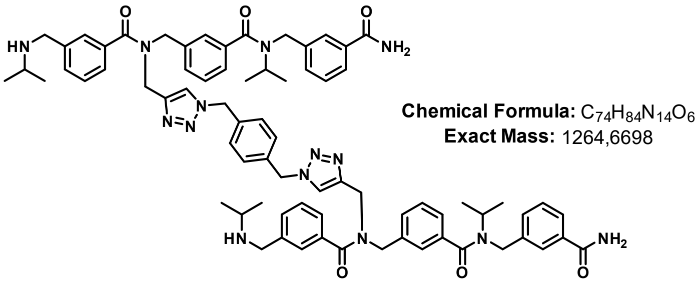

LCMS spectrum of the crude and HRMS data for compound **IV.1**:

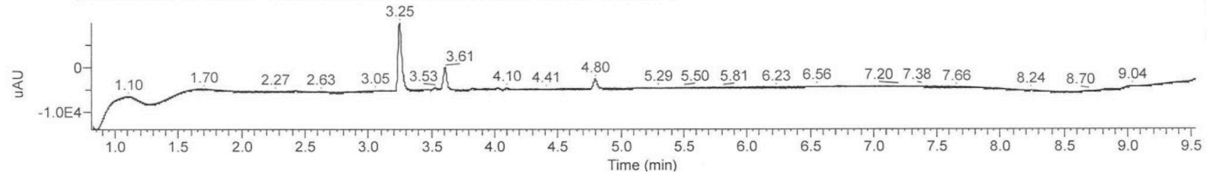

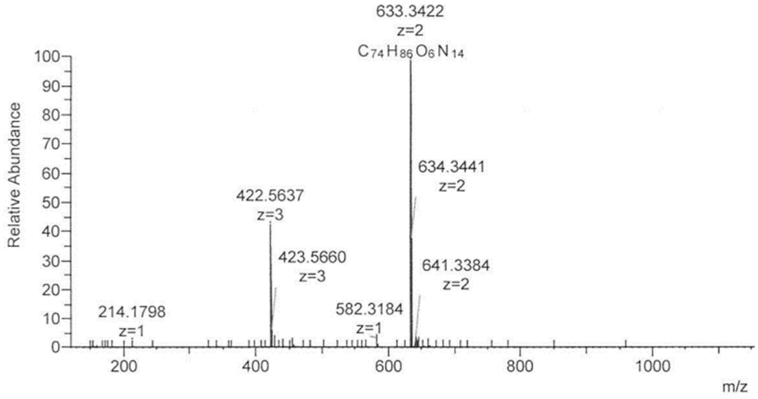

Peak Mass	Display Formula	Delta [ppm]	Theo. mass
633.3422	$C_{74}H_{86}O_6N_{14}$	0.05	633.34219

Copy of ^{1}H NMR spectrum of purified compound **IV.1** (400 MHz, $CDCl_3$):

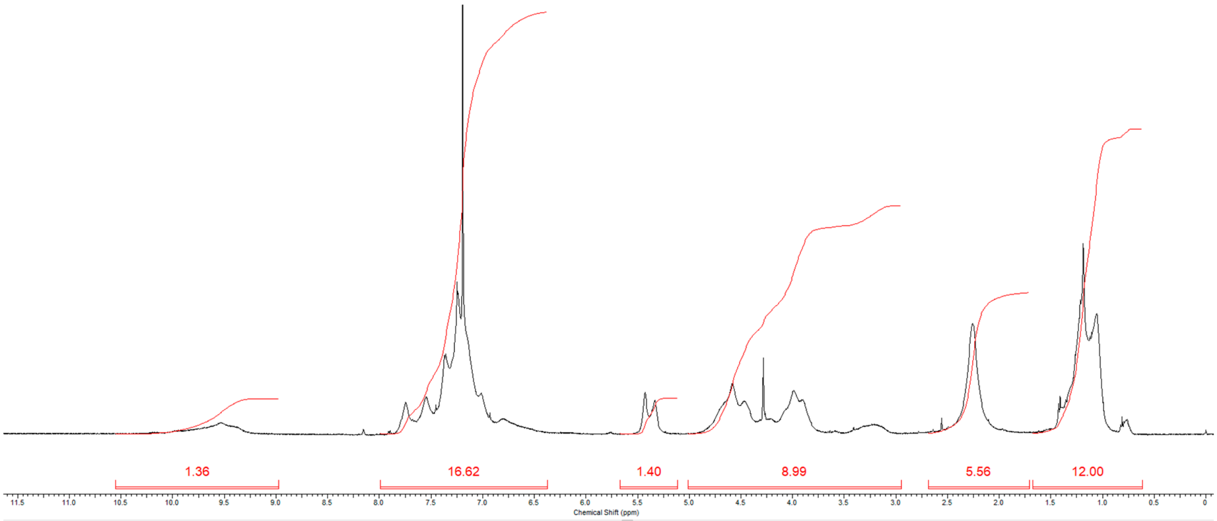

HPLC chromatogram and UV purity of purified compound **IV.1**:

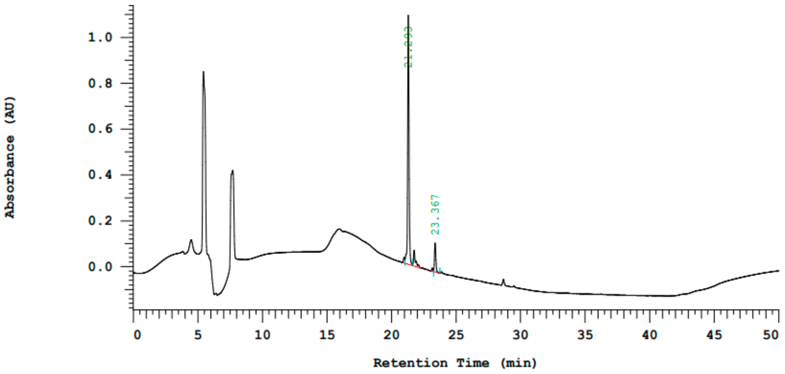

No.	RT	Area	Conc 1	BC
1	21.293	3844470	89.567	VV
2	23.367	447807	10.433	VB
		4292277	100.000	

Compound V:

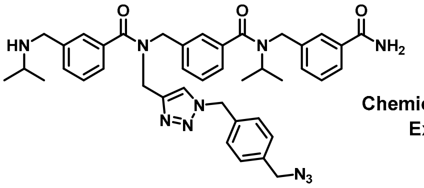

Chemical Formula: $C_{41}H_{46}N_{10}O_3$
Exact Mass: 726,3754

LCMS spectrum and HRMS data for purified compound **V**:

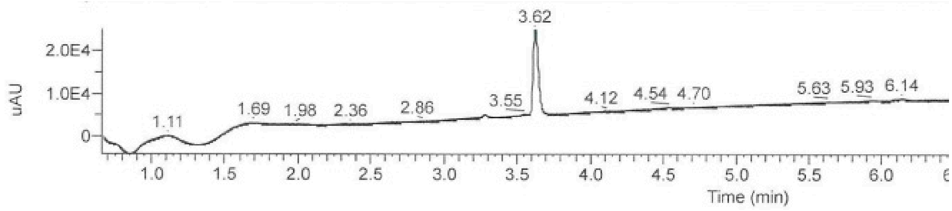

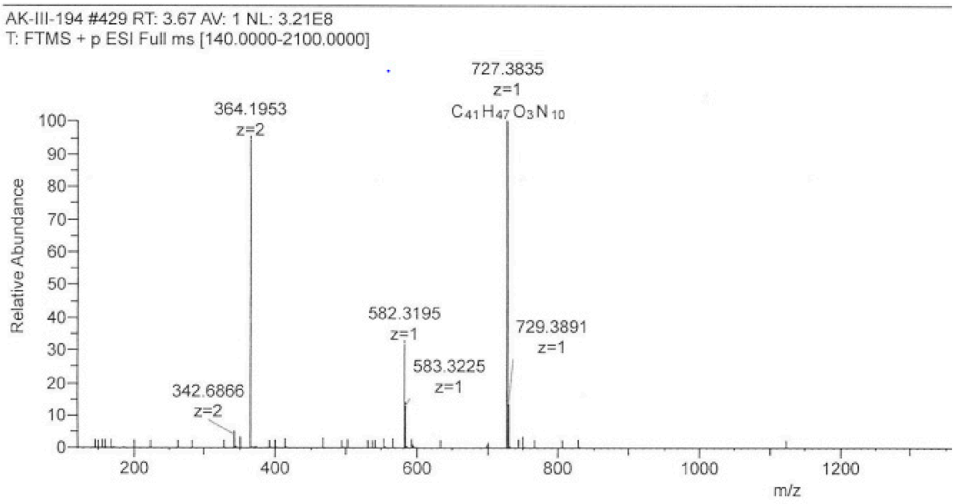

Peak Mass	Display Formula	Delta [ppm]	Theo. mass	Combined Score	Pattern Cov. [%]	MSMS Matched Fragments
727.3835	$C_{41}H_{47}O_3N_{10}$	1.15	727.38271	99.18	100	(Collection)

Copy of ^{1}H NMR spectrum of purified compound **V** (400 MHz, $CDCl_3$):

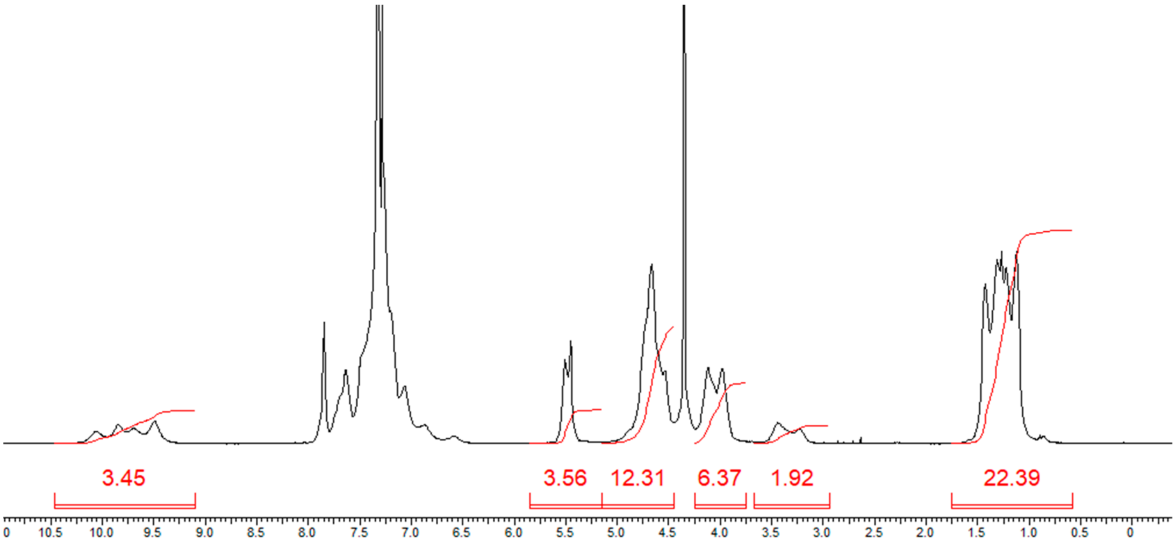

HPLC chromatogram and UV purity of purified compound **V**:

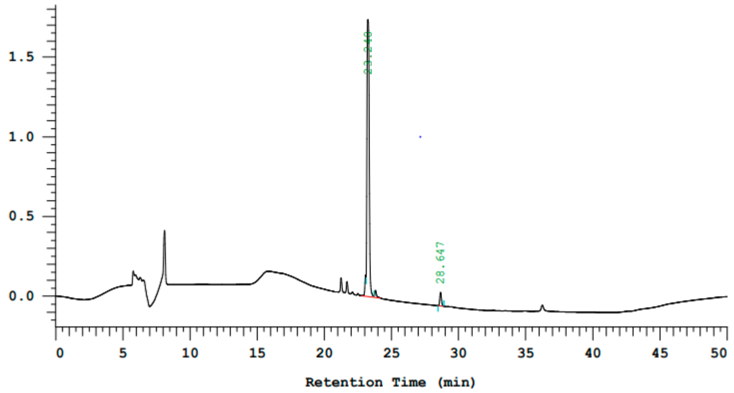

No.	RT	Area	Conc 1	BC
1	23.240	9824614	96.617	VV
2	28.647	343969	3.383	BB
		10168583	100.000	

Compound IV.3:

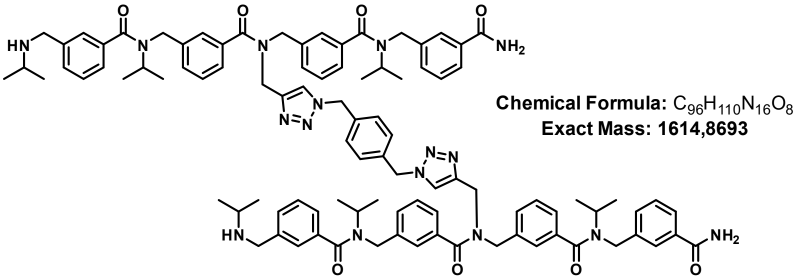

LCMS spectrum of the crude and HRMS data for compound **IV.3**:

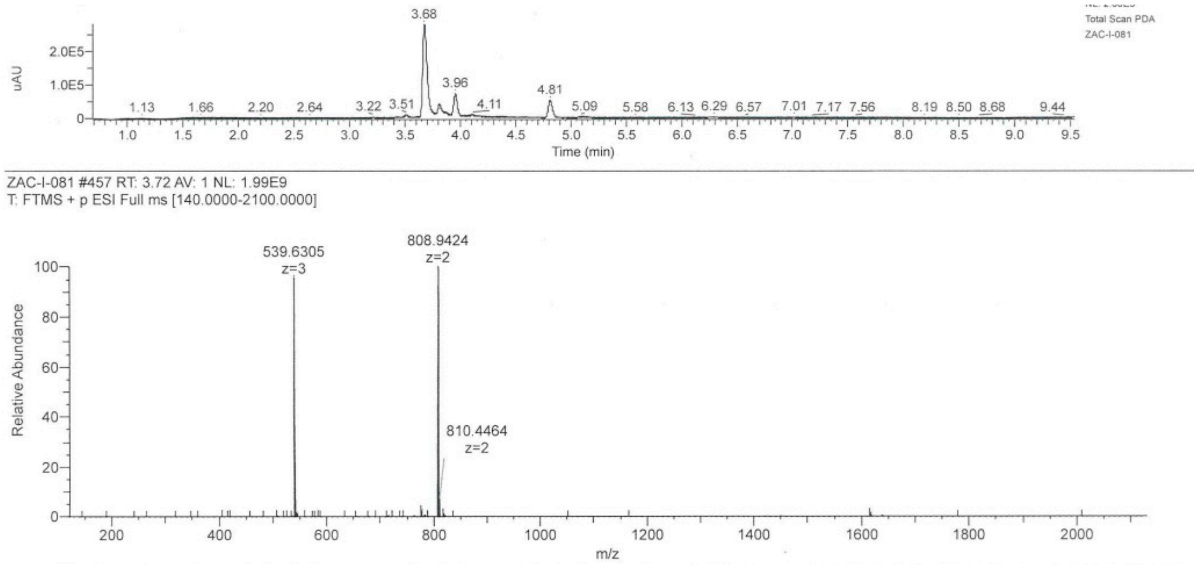

Peak Mass	Display Formula	Delta [ppm]	Theo. mass	Combined Score	Pattern Cov. [%]	MSMS Matched Fragments
808.4412	$C_{96}H_{112}O_8N_{16}$	-0.92	808.44190	98.46	100	(Collection)

Copy of ^{1}H NMR spectrum of purified compound **IV.3** (400 MHz, $CDCl_3$):

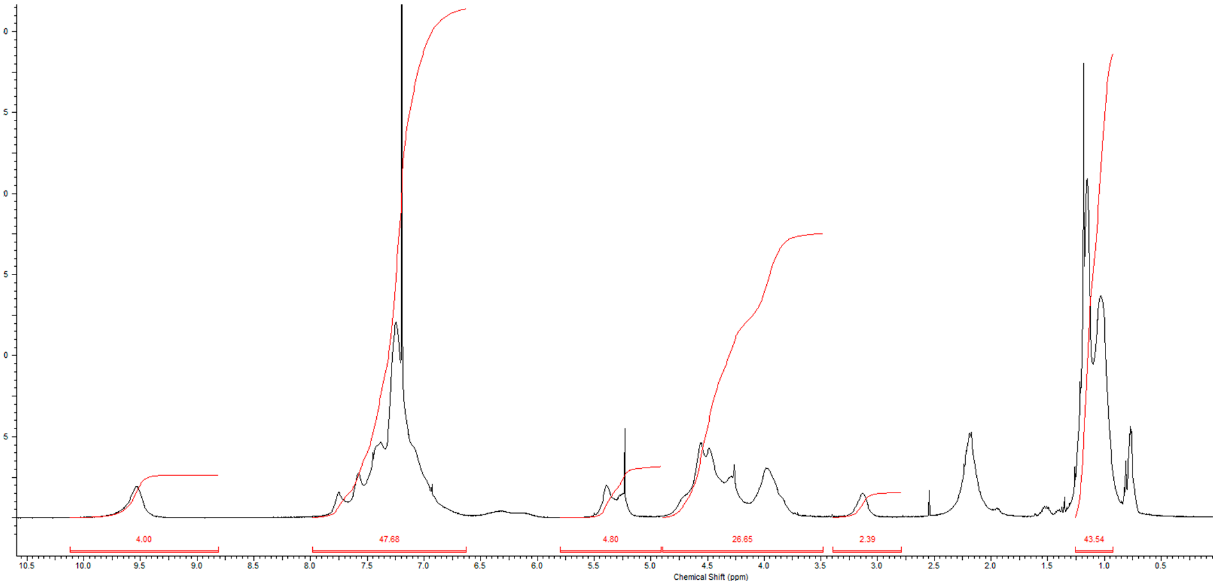

HPLC chromatogram and UV purity of purified compound **IV.3**:

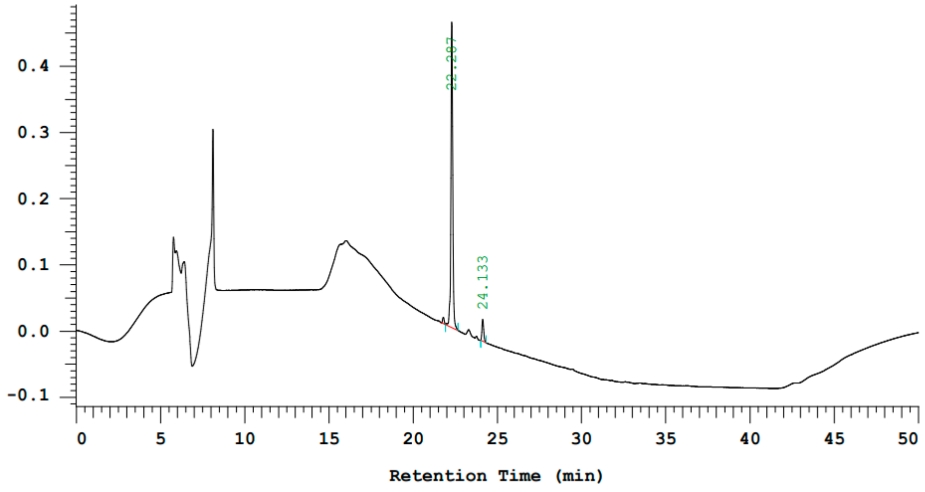

No.	RT	Area	Conc 1	BC
1	22.287	1624888	93.522	BB
2	24.133	112560	6.478	BB
		1737448	100.000	

Compound IV.4:

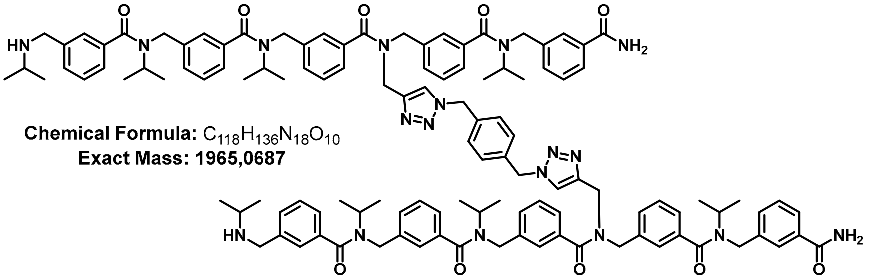

LCMS spectrum of the crude and HRMS data for compound **IV.4**:

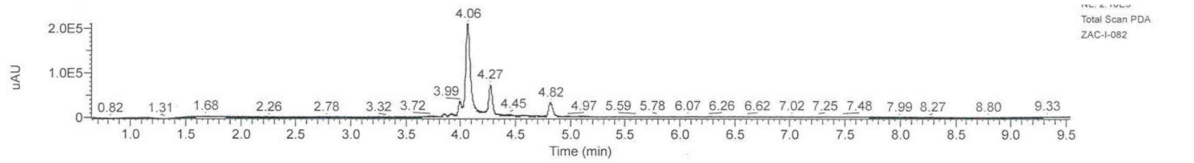

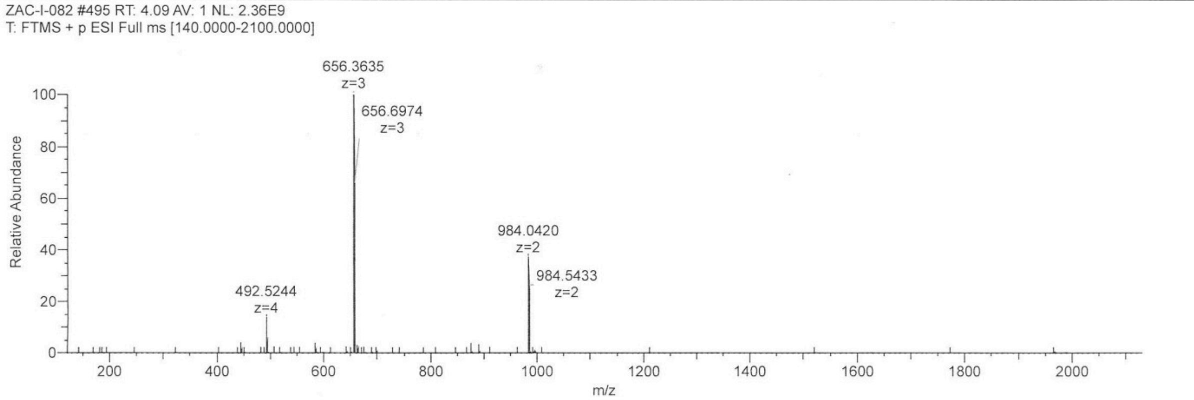

Peak Mass	Display Formula	Delta [ppm]	Theo. mass	Combined Score	Pattern Cov. [%]	MSMS Matched Fragments
983.5405	$C_{118}H_{138}O_{10}N_{18}$	-1.11	983.54162	98.15	100	(Collection)

Copy of ^{1}H NMR spectrum of purified compound **IV.4** (400 MHz, $CDCl_3$):

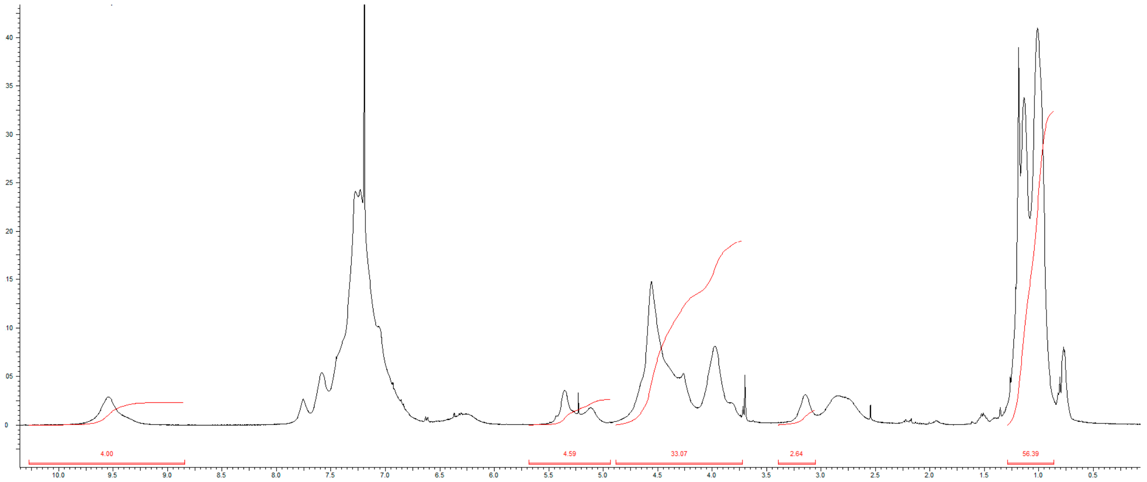

HPLC chromatogram and UV purity of purified compound **IV.4**:

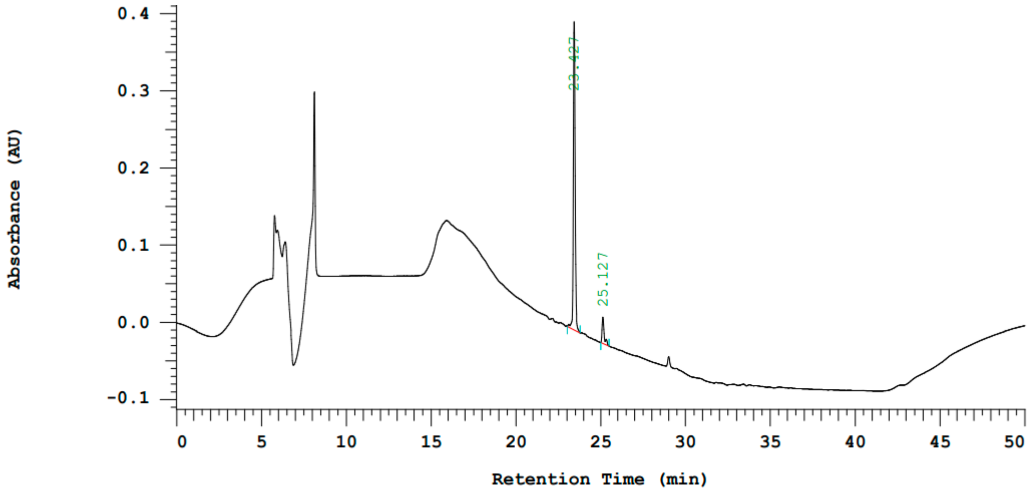

No.	RT	Area	Conc 1	BC
1	23.427	1385005	90.886	BB
2	25.127	138888	9.114	BB
		1523893	100.000	

Compound IV.5:

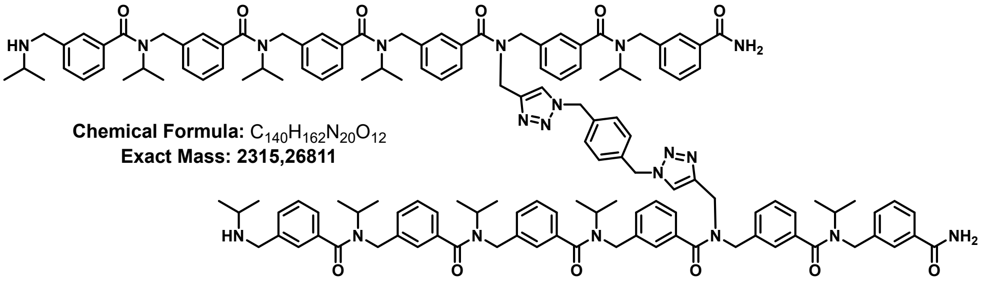

LCMS spectrum and HRMS data for compound **IV.5**:

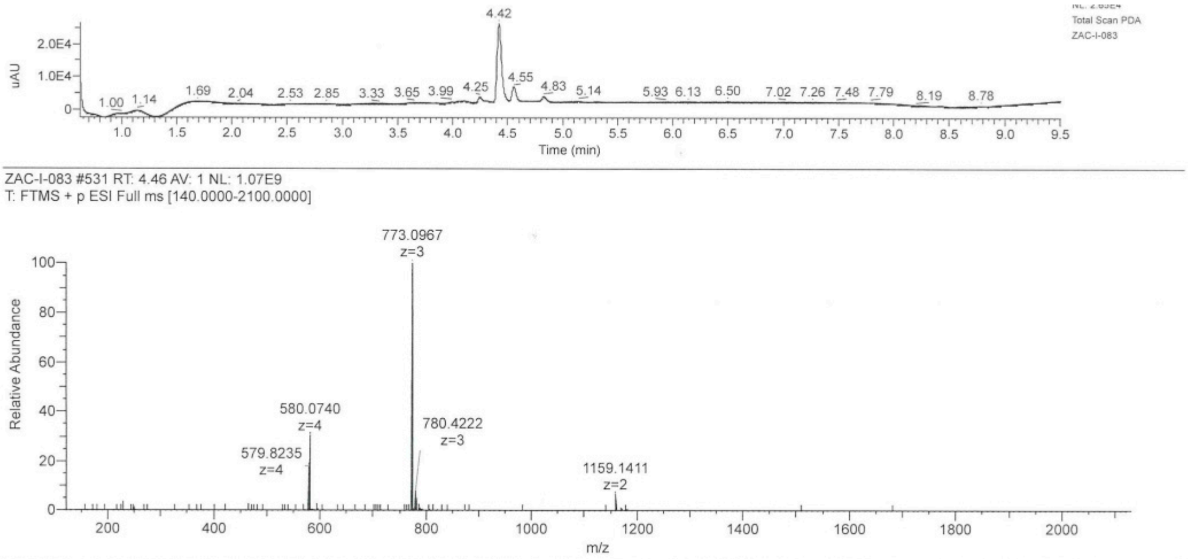

Peak Mass	Display Formula	Delta [ppm]	Theo. mass	Combined Score	Pattern Cov. [%]	MSMS Matched Fragments
772.7634	$C_{140}H_{165}O_{12}N_{20}$	0.15	772.76331	98.1	100	(Collection)

Copy of ^{1}H NMR spectrum of purified compound **IV.5** (400 MHz, $CDCl_3$):

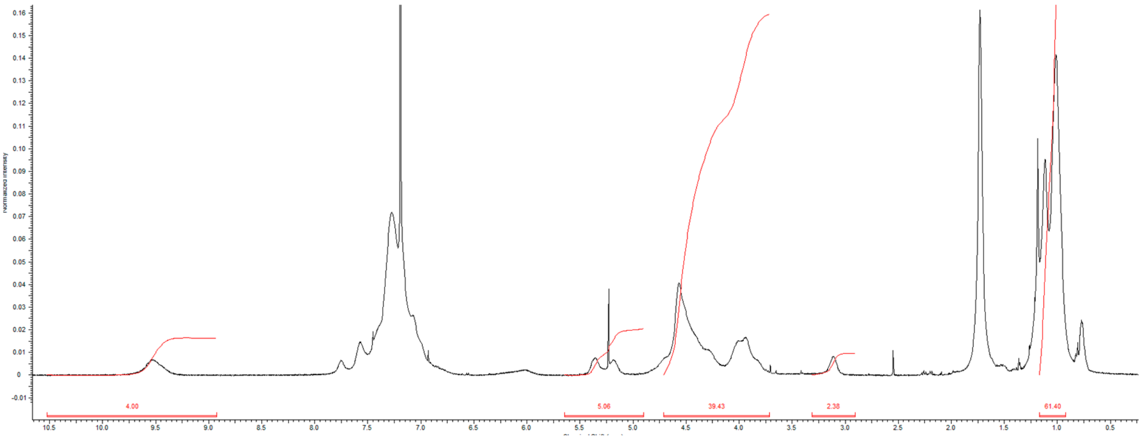

HPLC chromatogram and UV purity of purified compound **IV.5**:

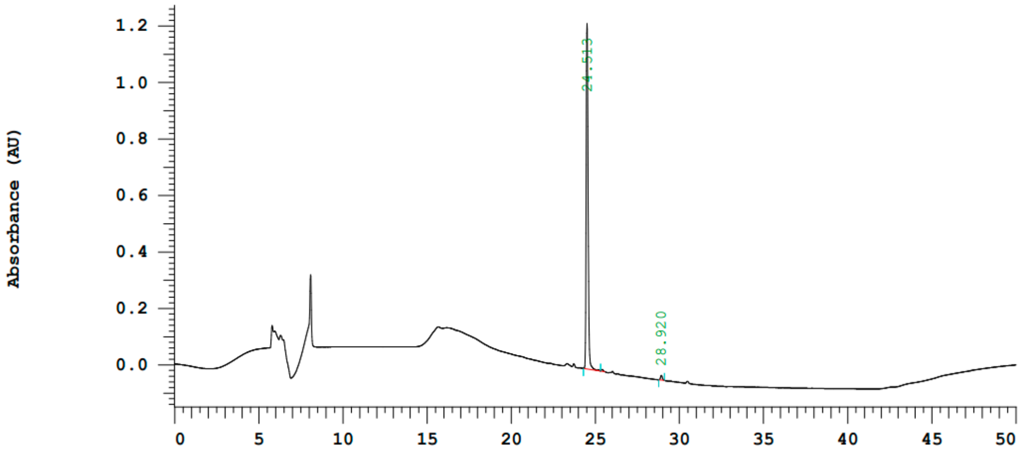

No.	RT	Area	Conc 1	BC
1	24.513	4489313	98.659	BB
2	28.920	61002	1.341	BB
		4550315	100.000	

Compounds IV.6:

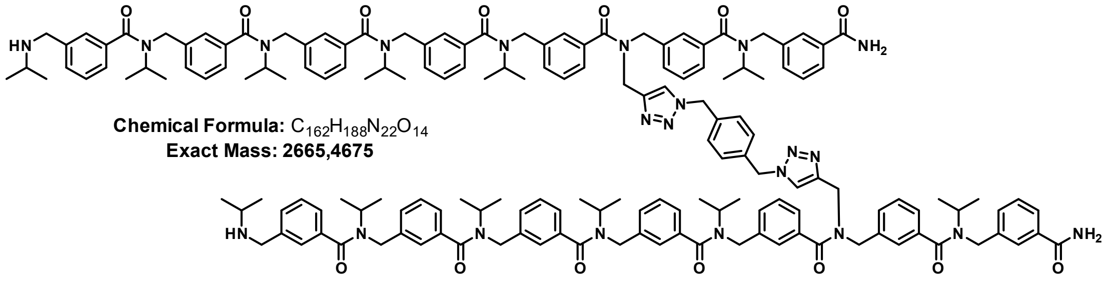

LCMS spectrum and HRMS data for compound **IV.6**:

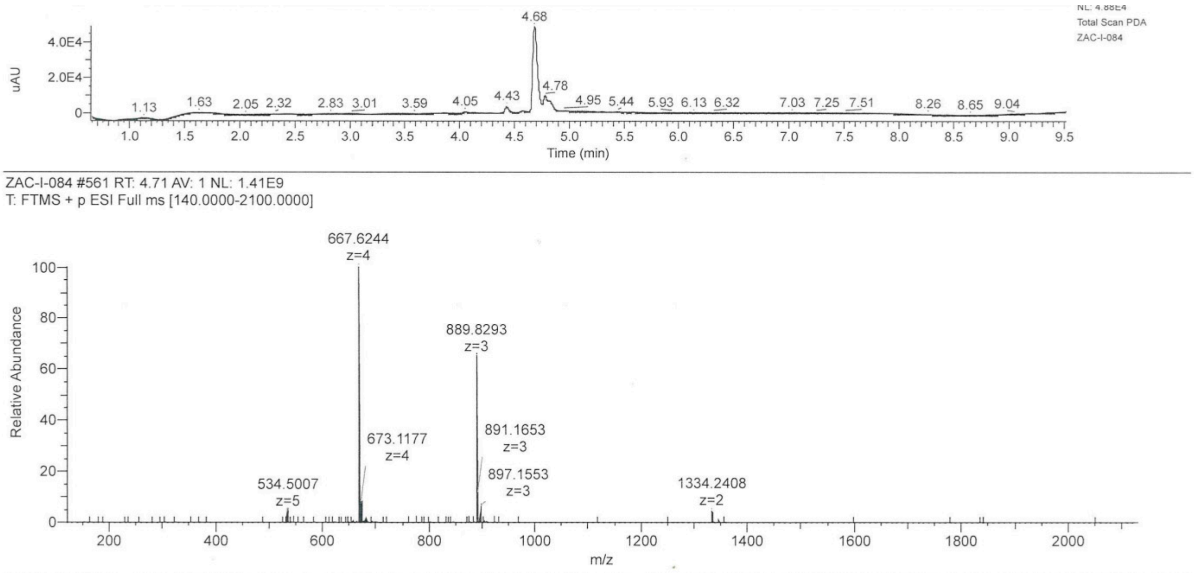

Peak Mass	Display Formula	Delta [ppm]	Theo. mass	Combined Score	Pattern Cov. [%]	MSMS Matched Fragments
889.4958	$C_{162}H_{191}O_{14}N_{22}$	-0.68	889.49646	96.58	99.65	(Collection)

Copy of ^{1}H NMR spectrum of purified compound **IV.6** (400 MHz, $CDCl_3$):

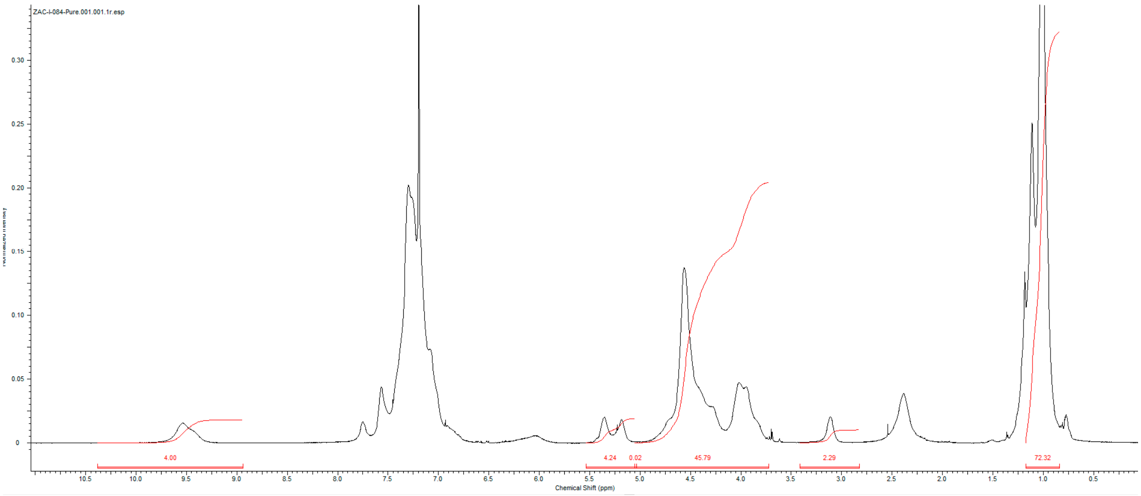

HPLC chromatogram and UV purity of purified compound **IV.6**:

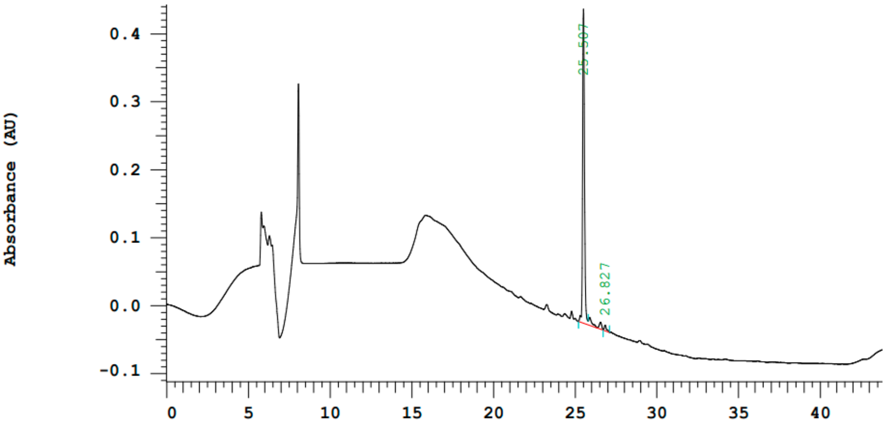

No.	RT	Area	Conc 1	BC
1	25.507	1725995	97.882	BV
2	26.827	37350	2.118	BB
		1763345	100.000	

Compounds IV.8:

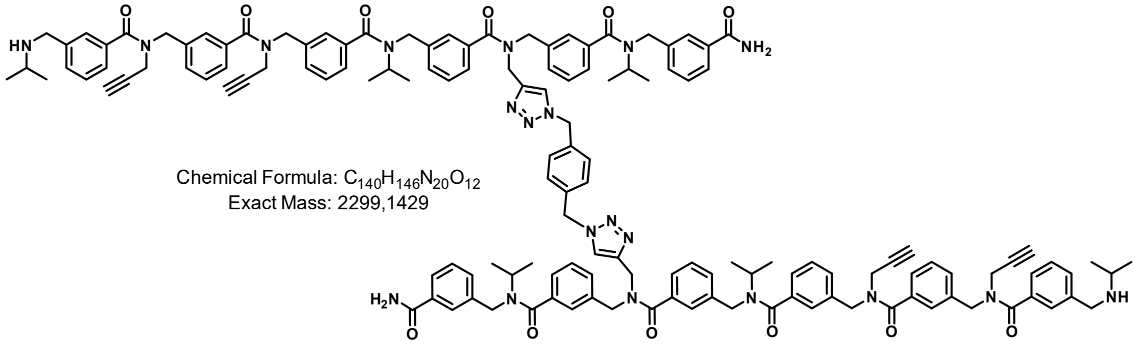

LCMS spectrum and HRMS data for compound **IV.8**:

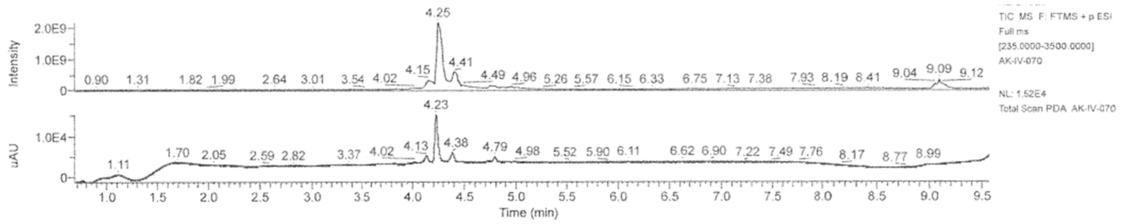

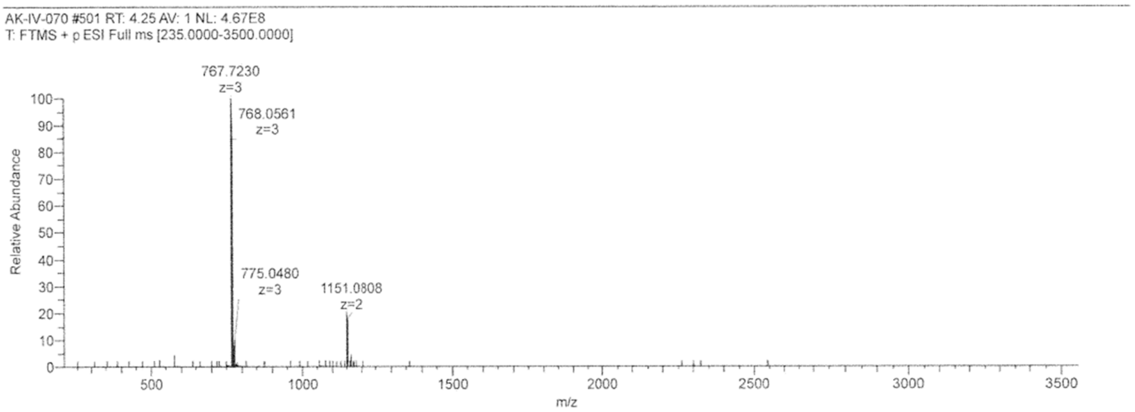

Peak Mass	Display Formula	Delta [ppm]	Theo. mass	Combined Score	Pattern Cov. [%]	MSMS Matched Fragments
767.3898	$C_{140}H_{149}O_{12}N_{20}$	1.99	767.38825	98.62	100	(Collection)

Copy of ^{1}H NMR spectrum of purified compound **IV.8** (400 MHz, $CDCl_3$):

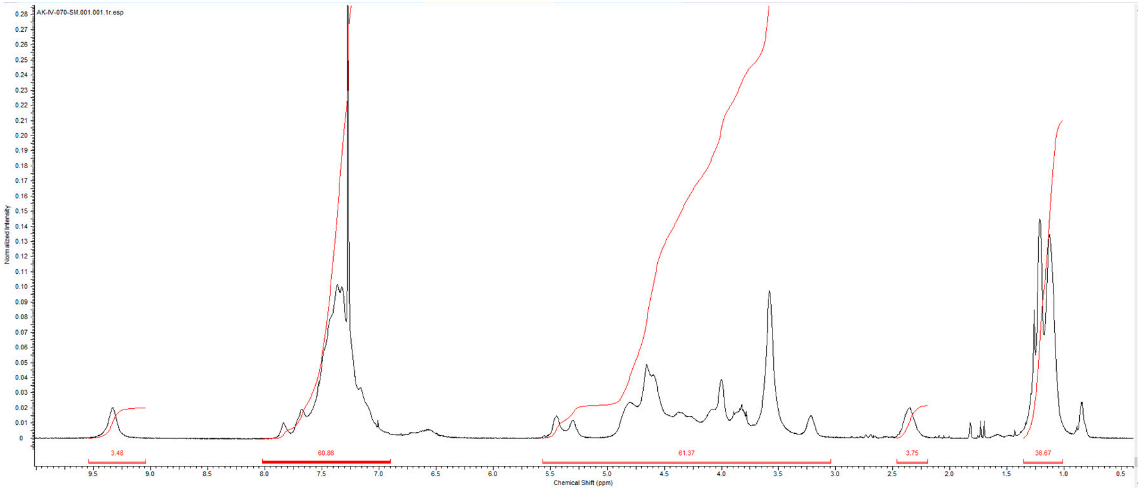

Compounds IV.9:

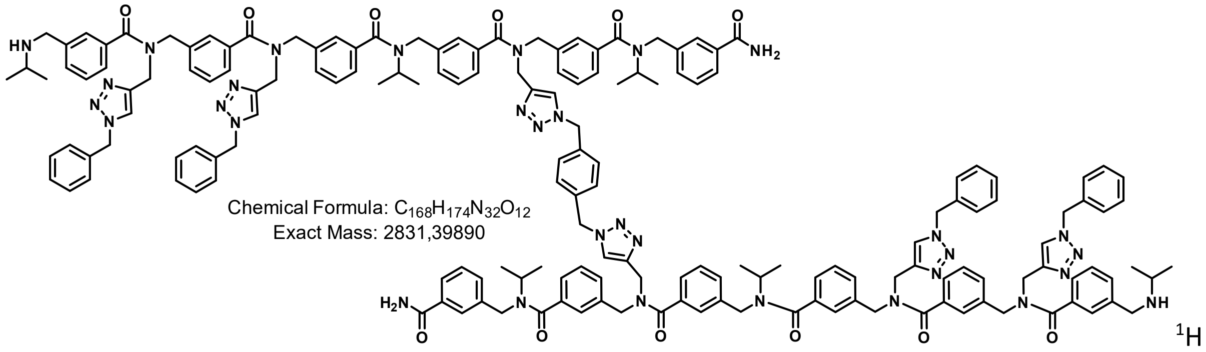

LCMS spectrum and HRMS data for compound **IV.9**:

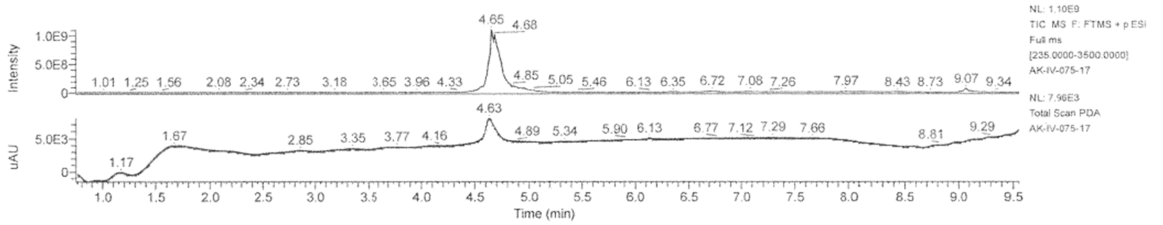

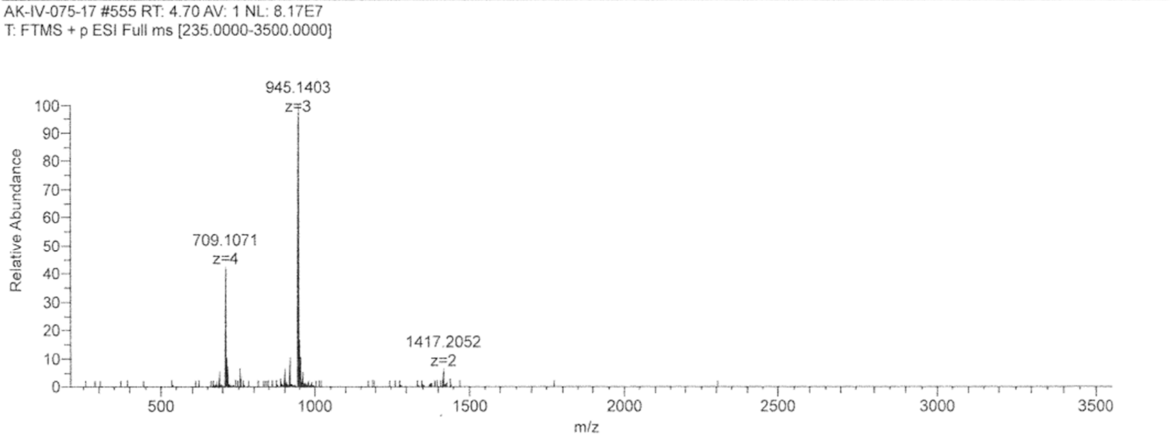

Peak Mass	Display Formula	Delta [ppm]	Theo. mass	Combined Score	Pattern Cov. [%]	MSMS Matched Fragments
944.8060	$C_{168}H_{177}O_{12}N_{32}$	-1.00	944.80691	78.64	85.68	(Collection)

Copy of ^{1}H NMR spectrum of purified compound **IV.9** (400 MHz, $CDCl_3$):

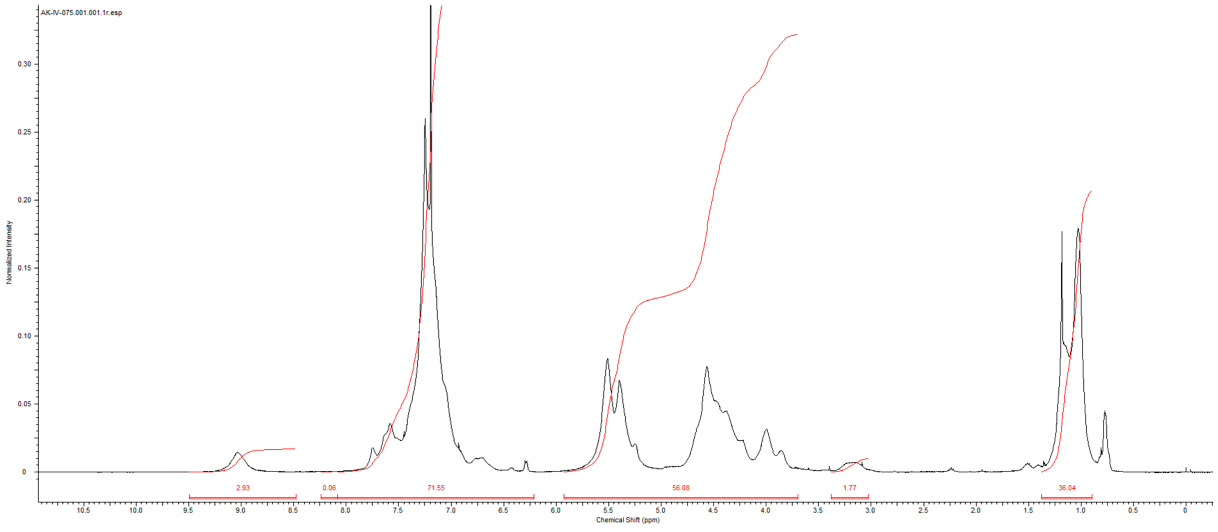

Compounds IV.10:

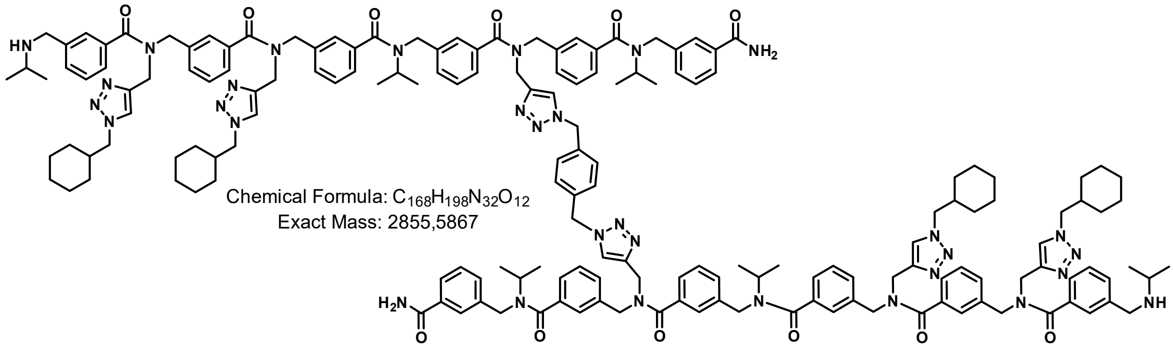

LCMS spectrum and HRMS data for compound **IV.10**:

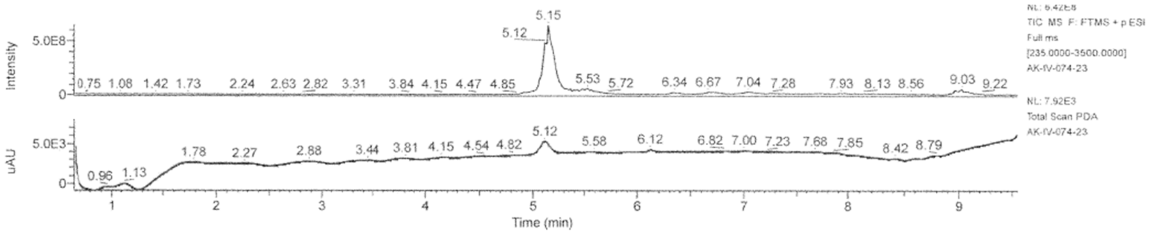

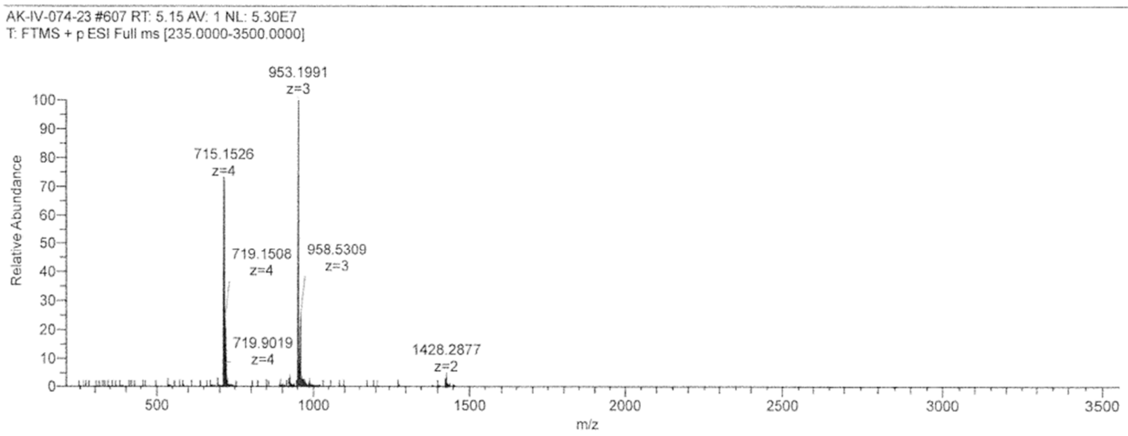

Peak Mass	Display Formula	Delta [ppm]	Theo. mass	Combined Score	Pattern Cov. [%]	MSMS Matched Fragments
714.9019	$C_{168}H_{202}O_{12}N_{32}$	-2.85	714.90395	84.29	85.83	(Collection)

Copy of ^{1}H NMR spectrum of purified compound **IV.10** (400 MHz, $CDCl_3$):

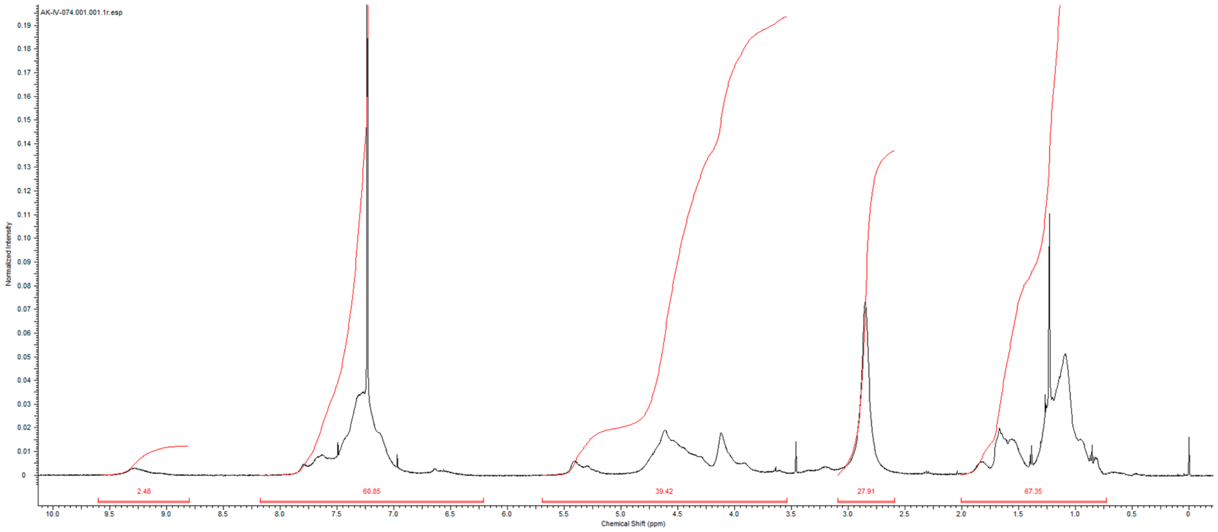

Supplement: Supplementary file 1 [file molecules-30-00724-s001.zip › molecules-3385050-supplementary.pdf]
